# The association between self-rated health and high-sensitivity C-reactive protein level: a cross-sectional and 5-year longitudinal study

**DOI:** 10.1186/s12889-018-6251-6

**Published:** 2018-12-17

**Authors:** Takashi Tamura, Mariko Naito, Kenta Maruyama, Mineko Tsukamoto, Tae Sasakabe, Rieko Okada, Sayo Kawai, Asahi Hishida, Kenji Wakai

**Affiliations:** 1 Department of Preventive Medicine, Nagoya University Graduate School of Medicine, 65 Tsurumai-cho, Showa-ku, Nagoya, 466-8550 Japan; 2 Department of Oral Epidemiology, Graduate School of Biomedical and Health Sciences, Hiroshima University, Hiroshima, Japan; 3 Department of Public Health, Aichi Medical University School of Medicine, Nagakute, Japan

**Keywords:** Self-rated health, High-sensitivity C-reactive protein, Low-grade inflammation, Cross-sectional study, Long-term longitudinal study, Japan

## Abstract

**Background:**

Although self-rated health (SRH) independently predicts mortality, the biological background of this association remains unexplained. This study aimed to examine the association between SRH and serum high-sensitivity C-reactive protein (hsCRP) level.

**Methods:**

Subjects were 899 participants aged 35–69 years (237 men and 662 women) in the Daiko Study, part of the Japan Multi-Institutional Collaborative Cohort Study. They were enrolled from 2008 to 2010. Of the subjects, 666 participated in a second survey 5 years later. Lifestyle factors and SRH were assessed using a self-administered questionnaire. Serum hsCRP level was measured using a latex-enhanced immunonephelometric assay. The association between SRH and serum hsCRP level was evaluated using a general linear model with covariates. We further longitudinally investigated whether higher serum hsCRP level at baseline predicts poor SRH after 5 years using an unconditional logistic regression model.

**Results:**

A higher serum hsCRP level was significantly associated with poor SRH at baseline after adjusting for covariates (*p* for trend = 0.023). The age- and sex-adjusted odds ratio and 95% confidence interval (CI) for poor SRH after 5 years was 1.45 (95% CI: 0.76–2.78) for the highest tertile compared with the lowest tertile of serum hsCRP level at baseline with a significant linear trend (*p* for trend = 0.033), although the risk increase disappeared after adjustment for other covariates.

**Conclusions:**

The present study demonstrated that poor SRH is cross-sectionally associated with higher serum hsCRP level. However, the longitudinal data did not support the relationship between serum hsCRP level at baseline and future SRH. Further longitudinal studies that include data on mortality and multiple inflammatory markers are warranted to elucidate the possible role of low-grade inflammation in the association between SRH and mortality risk.

## Background

Self-rated health (SRH) is an independent predictor of mortality and disease morbidity even after adjusting for potential confounders, such as medical risk factors, in the general population; the predictive value of SRH is surprisingly robust across different countries [1, 2]. The response from a single question on SRH (e.g., “In general, would you say that your health is excellent, very good, good, fair, or poor?”) is therefore considered to be as relevant as traditional objective risk factors to individual health status. However, the biological background of this association remains unexplained [3, 4]. Previous cross-sectional studies have shown that poor SRH is associated with mildly elevated serum high-sensitivity C-reactive protein (hsCRP) level, which is known to be a non-specific systemic inflammatory marker [5–12]. As low-grade inflammation plays an important role in the development of some diseases, such as diabetes mellitus, cardiovascular disease, and cancer [13–15], recent data suggest that the inflammatory response is a mediator of the association between poor SRH and increased risks of mortality and disease morbidity. Nevertheless, to our knowledge, only one study in Japan has examined the association between SRH and serum hsCRP level [9]. In addition, there have been a few long-term longitudinal studies investigating this relationship; three studies have performed a longitudinal analysis investigating biological predictors of changes in SRH, including CRP [6] or other biomarkers [16, 17].

The present study, therefore, aimed to examine i) whether SRH is cross-sectionally associated with serum hsCRP level and ii) whether serum hsCRP level predicts SRH after 5 years.

## Methods

### Subjects

Subjects of this study were participants in the Daiko Study, part of the Japan Multi-Institutional Collaborative Cohort Study (J-MICC Study). Nagoya city residents aged 35 to 69 years were invited to participate in the baseline survey using leaflets distributed in mailboxes citywide from 2008 to 2010. A total of 5172 residents provided written informed consent and completed a self-administered questionnaire on their lifestyle, including medical history. The design and rationale of the Daiko Study have been described in detail elsewhere [18]. Of the participants, 3543 individuals (68.5% of the participants in the baseline survey) participated in a second survey from 2014 to 2015.

The present cross-sectional study included 899 individuals (237 men and 662 women) with data on serum hsCRP level at baseline, after excluding those with serum hsCRP level of 1500 ng/mL or higher (*n* = 68) and those who had not provided medical history information (*n* = 13). We further conducted a 5-year longitudinal analysis with a cohort that consisted of those who had participated in both the baseline and the second survey to examine the relationship between SRH at the second survey and serum hsCRP level at baseline (*n* = 666, representing 74.1% of the initial cohort constituted of 899 participants). We additionally examined whether higher serum hsCRP level at baseline predicts incidence of poor SRH after 5 years among those without poor SRH at baseline who had participated in the second survey (*n* = 587).

The J-MICC Study and the Daiko Study were both approved by the ethics review committee of Nagoya University Graduate School of Medicine (approval numbers: 939 and 618, respectively).

### Assessment of lifestyle factors and SRH

A self-administered questionnaire was used that included the following demographic characteristics and lifestyle factors: alcohol consumption, smoking status, education level, sleeping hours, exercise habits, psychological stress, and medical history. Psychological stress was assessed using a single question: “Have you felt stress in the last year?” with the following four response options: “Not at all,” “Not much,” “A little,” and “High.” Height and weight were directly measured on the day of survey; body mass index (BMI) was calculated as weight in kilograms divided by the square of the height in meters (kg/m^2^). Physical activity was estimated as metabolic equivalents per day from the frequency and duration of daily and leisure time activities [19, 20]. Total energy intake was estimated in kilocalories per day using a validated food frequency questionnaire [21, 22].

SRH was assessed using a single question: “How do you rate your health in the last few months?” with the following five response options: “pretty good,” “rather good,” “neither good nor poor,” “rather poor,” and “pretty poor.” For the present analysis, those who answered “rather poor” or “pretty poor” were combined into a poor SRH group because of the small number of those who responded “pretty poor” at baseline (*n* = 9).

### Blood samples and measurement of serum hsCRP level

Peripheral blood was drawn from participants in the morning after overnight fasting. Serum hsCRP level was measured using a latex-enhanced immunonephelometric assay with a coefficient of variation less than 5%, using the Dade Behring Nephelometer II system at SRL Co., Ltd., Hachioji, Japan. The limit of detection (LOD) for the hsCRP assay was 50 ng/mL. A total of 52 subjects (5.8%) had serum hsCRP levels below the detectable value. We therefore substituted half the LOD value for values below the LOD [23]. Serum CRP has been reported to be a fairly stable biomarker with no observable circadian rhythm [24].

### Statistical analyses

Differences in mean values among SRH levels at baseline were examined by the one-way analysis of variance, and the differences in proportions were tested using the chi-square test. Least-squared means of serum hsCRP concentration according to SRH at baseline and the second survey were estimated using a general linear model with the following potential confounders: age (as a continuous variable), sex (for men and women combined only), alcohol consumption (never, former, current), smoking status (never, former, current), education level (≤9, 10–15, ≥16 years), BMI (as a continuous variable), physical activity (as a continuous variable), psychological stress (not at all, not much, a little, high), sleeping hours (as a continuous variable), medical history (yes, no; diabetes mellitus, dyslipidemia, hypertension, cardiovascular disease, stroke, and cancer), and energy intake (as a continuous variable). As a covariate, SRH at baseline (pretty good, rather good, neither good nor poor, poor) was additionally controlled in the longitudinal analysis. The linear trend between SRH and serum hsCRP level was examined using Scheffe’s test after assigning an ordinal score to each SRH group in the model. Participants with missing data for education level and psychological stress were included as additional categories in the analyses (*n* = 11).

Crude, age- and sex-adjusted, and multivariate-adjusted odds ratios (ORs) and 95% confidence intervals (CIs) of poor SRH after 5 years were estimated for tertile groups of serum hsCRP level at baseline using an unconditional logistic regression model with the above-mentioned covariates among those without poor SRH at baseline who participated in the second survey. The linear trend for risk was evaluated using the continuous value of serum hsCRP level.

A two-sided *p*-value of < 0.05 was considered statistically significant. All statistical analyses were performed using SAS 9.4 M5, which runs on SAS University Edition (SAS Institute Inc., Cary, NC, USA).

## Results

Table 1 shows characteristics of 899 subjects according to SRH level at baseline. Mean age ± standard deviation was 53.0 ± 10.1 years, and 26.4% of the participants were men. The proportions of current drinkers and smokers were 58.5 and 12.0%, respectively. Participants who had poor SRH were more likely to be young, women, nondrinkers, smokers, physically inactive, and stressed. There were no differences between SRH levels in the mean scores for BMI, sleeping hours, and energy intake, or in the distribution of education level and medical history.

**Table 1:** Characteristics of subjects by SRH level at baseline (*n* = 899)

Characteristics	SRH at baseline								
	Pretty good		Rather good		Neither good nor poor		Poor^a^		*p* for difference
No. of subjects (n, %)	98	(10.9)	252	(28.0)	430	(47.8)	119	(13.2)	
Age (years, mean ± SD)	52.5 ± 10.3		54.6 ± 10.4		52.7 ± 9.6		51.0 ± 10.5		0.009
Sex (men, n, %)	34	(34.7)	74	(29.4)	102	(23.7)	27	(22.7)	0.070
Alcohol consumption (n, %)									0.100
Never	30	(30.6)	94	(37.3)	175	(40.7)	60	(50.4)	
Former	2	(2.0)	3	(1.2)	8	(1.9)	1	(0.8)	
Current	66	(67.4)	155	(61.5)	247	(57.4)	58	(48.7)	
Smoking status (n, %)									0.063
Never	63	(64.3)	166	(65.9)	304	(70.7)	75	(63.0)	
Former	24	(24.5)	54	(21.4)	85	(19.8)	20	(16.8)	
Current	11	(11.2)	32	(12.7)	41	(9.5)	24	(20.2)	
Education level (years, n, %)									0.172
≤ 9	3	(3.1)	14	(5.6)	20	(4.7)	3	(2.5)	
10–15	56	(57.1)	148	(58.7)	286	(66.5)	85	(71.4)	
≥ 16	39	(39.8)	88	(34.9)	121	(28.1)	31	(26.1)	
Unknown	0	(0.0)	2	(0.8)	3	(0.7)	0	(0.0)	
Body mass index (kg/m^2^, mean ± SD)	21.2 ± 2.6		21.3 ± 2.8		21.4 ± 2.9		21.7 ± 3.4		0.575
Physical activity (METs · hours/day, mean ± SD)	16.2 ± 8.6		15.5 ± 9.8		14.5 ± 9.4		14.3 ± 10.0		0.258
Psychological stress (n, %)									< 0.001
Not at all	9	(9.2)	4	(1.6)	5	(1.2)	0	(0.0)	
Not much	22	(22.5)	50	(19.8)	45	(10.5)	7	(5.9)	
A little	43	(43.9)	132	(52.4)	222	(51.6)	36	(30.3)	
High	24	(24.5)	63	(25.0)	156	(36.3)	75	(63.0)	
Unknown	0	(0.0)	3	(1.2)	2	(0.5)	1	(0.8)	
Sleeping hours (hours, mean ± SD)	6.8 ± 0.9		6.7 ± 0.9		6.6 ± 0.9		6.6 ± 0.9		0.113
Medical history (current or former, n, %)									
Diabetes mellitus	1	(1.0)	9	(3.6)	13	(3.0)	5	(4.2)	0.557
Dyslipidemia	15	(15.3)	41	(16.3)	85	(19.8)	22	(18.5)	0.593
Hypertension	9	(9.2)	26	(10.3)	60	(14.0)	17	(14.3)	0.352
Cardiovascular disease	3	(3.1)	4	(1.6)	10	(2.3)	7	(5.9)	0.106
Stroke	1	(1.0)	2	(0.8)	6	(1.4)	1	(0.8)	0.889
Cancer	7	(7.1)	13	(5.2)	17	(4.0)	5	(4.2)	0.565
Energy intake (kcal/day, mean ± SD)	1627 ± 282		1632 ± 325		1590 ± 290		1595 ± 372		0.328

*Kcal* kilocalories, *METs* metabolic equivalents, *SD* standard deviation, *SRH* self-rated health

^a^The “Rather poor” and “Pretty poor” categories were combined into a “Poor” group because of the small number of those who responded “Pretty poor” at baseline (*n* = 9)

Crude means and least-squared means of serum hsCRP level according to SRH at baseline are shown in Table 2. Serum hsCRP crude means monotonically increased with poor SRH in the total population as well as in men and women (*p* for trend = 0.015, 0.033, and 0.050, respectively). The age- and sex- adjusted means ± standard errors (SE) (ng/mL) were 304.9 ± 28.2 for pretty good, 322.9 ± 18.1 for rather good, 349.9 ± 14.5 for neither good nor poor, and 418.1 ± 26.1 for poor (*p* for trend = 0.001). The analysis of the least-squared means with other covariates also demonstrated that poor SRH at baseline was significantly associated with a higher serum hsCRP level in the total population (*p* for trend = 0.023); however, the analysis stratified by sex showed no significant association (*p* for trend = 0.161 for men and 0.234 for women). The relationships between SRH at the second survey and serum hsCRP level at baseline are summarized in Table 3 (*n* = 666). Poor SRH at the second survey was significantly correlated with a higher serum hsCRP level at baseline (*p* for trend = 0.025). In the analysis stratified by sex, the positive relationship was statistically significant for women, but not for men (*p* for trend = 0.028 and 0.201, respectively) although the direction of relationship was not different by sex. The age- and sex- adjusted means ± SE were 288.4 ± 33.2 for pretty good, 328.3 ± 20.0 for rather good, 350.7 ± 17.1 for neither good nor poor, and 412.3 ± 29.0 for poor (*p* for trend = 0.003). The relationship was not significant in the multivariate-adjusted model.

**Table 2:** Baseline means for hsCRP by SRH level at baseline (*n* = 899)

hsCRP (ng/mL)	SRH at baseline				
	Pretty good	Rather good	Neither good nor poor	Poor^b^	*p*_for trend_
No. of subjects	98	252	430	119	
Crude mean ± SD					
Men and women (*n* = 899)	288.6 ± 264.8	314.5 ± 272.0	325.4 ± 282.6	382.5 ± 343.3	0.015
Men (*n* = 237)	330.4 ± 224.2	385.4 ± 280.4	394.0 ± 266.5	486.0 ± 359.7	0.033
Women (*n* = 662)	266.5 ± 283.1	285.1 ± 263.6	304.1 ± 284.5	352.1 ± 334.4	0.050
Least-squared mean^a^ ± SE					
Men and women (*n* = 899)	473.9 ± 74.5	479.7 ± 72.2	498.3 ± 71.4	553.1 ± 74.2	0.023
Men (*n* = 237)	385.5 ± 129.8	419.6 ± 126.8	419.0 ± 125.6	497.1 ± 136.4	0.161
Women (*n* = 662)	451.4 ± 95.4	435.5 ± 92.9	448.1 ± 92.3	499.5 ± 94.3	0.234

^a^Adjusted for age (as a continuous variable), sex (for men and women combined only), alcohol consumption, smoking status, education level, body mass index (as a continuous variable), physical activity (as a continuous variable), psychological stress, sleeping hours (as a continuous variable), medical history, and energy intake (as a continuous variable)

^b^The “Rather poor” and “Pretty poor” categories were combined into a “Poor” group because of the small number of those who responded “Pretty poor” at baseline (*n* = 9)

*hsCRP* high-sensitivity C-reactive protein, *SD* standard deviation, *SE* standard error, *SRH* self-rated health

**Table 3:** Baseline means for hsCRP by SRH level at second survey (*n* = 666)

hsCRP (ng/mL)	SRH at second survey				
	Pretty good	Rather good	Neither good nor poor	Poor^b^	*p*_for trend_
No. of subjects	69	199	304	94	
Crude mean ± SD					
Men and women (n = 666)	270.9 ± 195.0	313.3 ± 263.9	327.1 ± 298.0	368.5 ± 318.7	0.025
Men (*n* = 173)	352.6 ± 218.5	368.7 ± 257.3	433.8 ± 292.9	433.6 ± 357.0	0.201
Women (*n* = 493)	232.7 ± 172.4	289.5 ± 264.0	295.8 ± 292.7	348.6 ± 306.0	0.028
Least-squared mean^a^ ± SE					
Men and women (*n* = 666)	544.0 ± 91.9	546.8 ± 87.0	570.2 ± 86.1	605.1 ± 88.5	0.117
Men (*n* = 173)	391.1 ± 162.6	386.4 ± 150.6	461.9 ± 143.9	492.7 ± 143.9	0.188
Women (*n* = 493)	553.4 ± 118.2	548.0 ± 114.3	555.9 ± 114.6	596.0 ± 117.3	0.381

^a^Adjusted for age (as a continuous variable), sex (for men and women combined only), alcohol consumption, smoking status, education level, body mass index (as a continuous variable), physical activity (as a continuous variable), psychological stress, sleeping hours (as a continuous variable), medical history, energy intake (as a continuous variable), and SRH at baseline

^b^The “Rather poor” and “Pretty poor” categories were combined into a “Poor” group because of the small number of those who responded “Pretty poor” at second survey (*n* = 14)

*hsCRP* high-sensitivity C-reactive protein, *SD* standard deviation, *SE* standard error, *SRH* self-rated health

We further longitudinally investigated whether higher serum hsCRP level at baseline predicts poor SRH after 5 years among those without poor SRH at baseline who participated in the second survey (*n* = 587). Table 4 shows the ORs and 95% CIs for poor SRH after 5 years in relation to serum hsCRP level at baseline. The age- and sex-adjusted ORs increased with higher serum hsCRP level; the ORs were 1.25 (0.67–2.34) for the middle tertile and 1.45 (0.76–2.78) for the highest tertile compared with the lowest tertile of serum hsCRP level with a significant trend (*p* for trend = 0.033). However, the crude and the multivariate-adjusted models showed no relationship.

**Table 4:** Poor SRH after 5 years and baseline hsCRP among those without poor SRH at baseline (*n* = 587)

	hsCRP			
	Lowest tertile	Middle tertile	Highest tertile	*p*_for trend_
hsCRP, range (ng/mL)	25–137	140–359	360–1380	
hsCRP, median (ng/mL)	84	226	549	
No. of subjects	193	198	196	
No. of new poor SRH cases^a^	22	24	24	
Crude OR (95% CI)	ref.	1.07 (0.58–1.99)	1.09 (0.59–2.01)	0.203
Age and sex-adjusted OR (95% CI)	ref.	1.25 (0.67–2.34)	1.45 (0.76–2.78)	0.033
Multivariate-adjusted OR^b^ (95% CI)	ref.	0.99 (0.51–1.94)	1.10 (0.54–2.25)	0.241

^a^Those who answered “Rather poor” or “Pretty poor” after 5 years

^b^Adjusted for age (as a continuous variable), sex, alcohol consumption, smoking status, education level, body mass index (as a continuous variable), physical activity (as a continuous variable), psychological stress, sleeping hours (as a continuous variable), medical history, energy intake (as a continuous variable), and SRH at baseline

*CI* confidence interval, *hsCRP* high-sensitivity C-reactive protein, *OR* odds ratio, *SRH* self-rated health

## Discussion

In this cross-sectional study, we found that a higher serum hsCRP level was associated with poor SRH in all the subjects. This significant association remained after adjustment for covariates including medical history, although the analysis stratified by sex showed no significant association. Poor SRH at the second survey was also associated with a higher serum hsCRP level at baseline; however, this relationship was not significant in the multivariate-adjusted model. The longitudinal analysis of participants without poor SRH at baseline showed that a higher serum hsCRP level at baseline was unrelated to poor SRH after 5 years after multivariate adjustment.

The present cross-sectional findings are in accord with findings from previous studies [5–12]. We found that poor SRH at the second survey was also associated with a higher serum hsCRP level at baseline, albeit not significantly, even after controlling for multiple confounders. These findings provide further evidence for an association between SRH and serum hsCRP level. Poor SRH is associated with several symptoms, such as fatigue, muscle ache, and reduced appetite [25–27], and these are typically induced by proinflammatory cytokines, such as interleukin 6, which stimulates the production of CRP in the liver [28]. As several studies have demonstrated that poor SRH is positively correlated with proinflammatory cytokine levels [5–8, 17, 29], the association between poor SRH and higher serum hsCRP level observed in the present study may be partially explained by cytokine-induced sickness behavior; however, we were unable to perform further analyses of the associations because we had no data for these markers or their associated symptoms. According to previous reports, plasma proinflammatory cytokine levels may be moderately correlated with serum hsCRP level [7, 8].

It is also well known that low-grade inflammation is associated with an increased risk of all-cause mortality [30–33]. The present study showed that low-grade inflammation, expressed as serum hsCRP level, was associated with poor SRH. Interestingly, a recent study has shown that low-grade inflammation, expressed as erythrocyte sedimentation rate, was also associated with poor SRH [34]. To elucidate the role of low-grade inflammation in the association between SRH and mortality, it is necessary to clarify whether poor SRH is associated with an increased risk of mortality even after adjusting for inflammatory marker levels or stratifying participants according to the presence of low-grade inflammation.

We were interested in whether serum hsCRP level predicts future SRH, as it is useful to clarify whether low-grade inflammation is associated with poor SRH. We therefore longitudinally investigated whether higher serum hsCRP level at baseline predicted poor SRH after 5 years among those without poor SRH at baseline who participated in the second survey. The risk of poor SRH after 5 years was significantly associated with higher serum hsCRP level at baseline in the age- and sex-adjusted model; however, the ORs were close to unity in the crude and the multivariate-adjusted model. A previous study in Sweden examining predictors of SRH after 1 year also found that serum hsCRP level was not associated with future SRH [6]. Thus, the predictive value of serum hsCRP level for future SRH may not be large, even though we observed a significant cross-sectional association; however, the limitations of relevant studies mean that its potential predictive value should not be ruled out. To the best of our knowledge, this is the first study to carry out both cross-sectional and 5-year longitudinal analyses on the association between SRH and serum hsCRP level in the same population. Cross-sectional findings including previous studies suggest that serum hsCRP level at baseline may be related to future SRH; however, we did not observe a clear consistent relationship in a longitudinal analysis after adjustment for covariates as well as a previous one [6]. Further longitudinal studies considering a change in serum hsCRP level may account for this inconsistency.

The strengths of the present study include its longitudinal design and the control of several confounding factors. An important limitation was that we did not include mortality data and data for other inflammatory markers. We were therefore unable to directly examine the extent to which SRH and low-grade inflammation are independently responsible for mortality, and the association of SRH with other inflammatory markers. Longitudinal research on SRH, multiple inflammatory markers, and mortality within the same population are needed. Sample size in this study was relatively small, particularly for men, resulting in low statistical power; however, we demonstrated consistent associations between SRH and serum hsCRP level for men and women separately, and these findings were similar to those of a previous study [9].

## Conclusions

In conclusion, poor SRH was cross-sectionally associated with a higher serum hsCRP level in the total population after adjustment for covariates although the analysis stratified by sex showed no significant association. The longitudinal analysis demonstrated no relationship between serum hsCRP level at baseline and future SRH. Further longitudinal studies that include data on mortality and multiple inflammatory markers are warranted to elucidate the possible role of low-grade inflammation in the association between SRH and mortality risk among the general population.

## Abbreviations

BMI: Body mass index
CI: Confidence interval
hsCRP: High-sensitivity C-reactive protein
J-MICC: Japan Multi-Institutional Collaborative Cohort
LOD: Limit of detection
OR: Odds ratio
SE: Standard error
SRH: Self-rated health
